# Is the urinary iodine/creatinine ratio applicable to assess short term individual iodine status in Chinese adults? Comparison of iodine estimates from 24-h urine and timed-spot urine samples in different periods of the day

**DOI:** 10.1186/s12986-022-00656-6

**Published:** 2022-04-07

**Authors:** Zhuan Liu, Yixuan Lin, Jiani Wu, Diqun Chen, Xiaoyan Wu, Ying Lan, Zhihui Chen

**Affiliations:** 1 The Department of Endemic Diseases, Fujian Center for Disease Control and Prevention Fujian, No. 386 Chong’an Road, Fuzhou, 350012 Fujian People’s Republic of China; 2 The Department of Disease Control and Prevention, The First Hospital of Quanzhou Affiliated to Fujian Medical University, No.248-252, Dongjie Road, Quanzhou, 362000 Fujian People’s Republic of China; 3 School of Public Health, Fujian Medical University, University of New Area, No.1 Xueyuan Road, Fuzhou, 350122 Fujian People’s Republic of China

**Keywords:** Urinary iodine/creatinine ratio, Individual iodine status, Urinary iodine excretion, Timed-spot urine

## Abstract

**Background:**

Urinary iodine concentration (UIC) is routinely used to evaluate the population iodine status while the uniform method for the individual level assessment is uncertain.

**Objectives:**

To explore the 24-h urinary iodine excretion (UIE) in five different periods of the day and the corresponding prediction equations respect by the use of creatinine-corrected UIC.

**Methods:**

We collected 24-h, spot and fasting urine in five periods of the day to estimate 24-h UIE by the six different prediction equations. We compared the estimated creatinine-corrected UIC to the collected 24-h UIE and identified the most suitable equations in each period of the day.

**Results:**

Among the six different prediction equations, the equation of Kawasaki T was the best to estimate the 24-h UIE by fasting urine among Chinese adults. Among the five periods of time, the equation of Knudsen N was the best to estimate the 24-h UIE in the non-morning period.

**Conclusion:**

Urinary iodine status at the individual level could be estimated by different creatinine-based equations at different periods of the day.

## Background

Iodine is a trace element that is demanded by the human body. However, iodine deficiency has multiple adverse effects on human growth and development [1], causing goitre and increasing the thyroid gland susceptibility to nuclear radiation in people of all ages. It can lead to abortion and premature birth in fetuses, endemic cretinism in neonate, and impaired mental function and delayed physical development in children [2]. For adults, especially the elderly, it can cause hyperthyroidism and hypothyroidism [3]. Iodine deficiency is regarded as a public health issue in both developing and developed countries, with an estimated number of 1.90 billion people worldwide at risk of insufficient iodine intake [4], especially in the Eastern Mediterranean, Asia, African and most of the Eastern European countries [5]. Iodine deficiency is a risk factor for most thyroid disorders, according to a recent epidemiological study conducted among Chinese adults whose median urinary iodine concentration (mUIC) were less than 100ug/L [6]. This study showed that the prevalence of subclinical hyperthyroidism was 0.34 percent among iodine deficiency population, for Graves' disease was 0.83 percent, for subclinical hypothyroidism was 11.62 percent, for goiter was 1.58 percent and for thyroid nodule was 25.63 percent [6]. In another study regardingt of iodine status on pregnancy outcomes, miscarriage (with a prevalence of 6.2% among iodine deficiency population), placental the impacabruption (with a prevalence of 1.09% among iodine deficiency population), and neonatal cord entanglement (with a prevalence of 1.82% among iodine deficiency population) were all found to be more common among early pregnancy women with iodine deficiency than in those with other iodine status [7]. Excessive iodine consumption, on the other hand, can cause thyroid diseases. Excess iodine was linked to a higher prevalence of overt hyperthyroidism and subclinical hypothyroidism (with a prevalence of 1.22% and 15.99% among the excessive iodine intake people, respectively) [6]. An increased incidence of autoimmune thyroiditis (AT) and hypothyroidism can also be caused by much iodine consumption [8].

Iodine, as a nutrient demanded for thyroid hormone synthesis [2], has an impact on body functions, such as brain development, pituitary support, and metabolism. The average amount of iodine within a human body is around 20 mg [9]. The body's iodine reserves only last for a period of time if iodine intake discontinues. The majority of iodine ingested by the human body comes from food, the remaining part comes from drinking water and the air. Due to the scarcity of iodine-rich foods [10], World Health Organization (WHO) introduced a salt iodization program to improve iodine status at the populational level as a way of lowering the risk of iodine insufficiency [11]. China [12], as well as most other countries [13, 14] has considerably improved this issue by the universal salt iodization (USI) program [15]. Iodised salt has become the primary source of iodine for the human body in the countries that have adopted the USI program. The limited dietary salt intake is recommended by the guidelines to prevent hypertension [16] which is a long-standing concern as a major cause of death and the second leading cause of disability [17]. However, a low-salt diet inhibits the amount of intake of iodine, which increases the risk of iodine deficiency in hypertension patients. In order to identify people who are at risk of iodine deficiency, it is critical to trace iodine status at both population and the individual level [18, 19].

More than 90% of dietary iodine is absorbed in the duodenum and excreted in urine via kidneys within 24–48 h [20]. The remaining is excreted in the feces, as well as in the lungs, skin, and mucous membranes to a lesser extent, and even in the breasts during lactation. And therefore, urinary iodine concentration (UIC) is applied in the assessment of recent iodine intake. Iodine excretion in the urine sample collected at the time point is often expressed as UIC or the urinary iodine/creatinine concentration ratio [21]. However, the spot urine UIC is limited by the daily variations of iodine intake and water intake, resulting in an uncorrected spot urine UIC that does not objectively reflect the actual individual iodine status and can therefore only be used to assess the iodine status of a population [14, 22]. Individual iodine status assessment methods are still in the exploration phase. Some researchers used serum iodine [23], serum thyroglobulin [24] and 24-h urine [25] to assess individual iodine status. Given that spot urine samples are easier to collect than other indicators, and 24-h urine can more objectively reflect individual iodine status, we compared the six creatinine-corrected spot urine UIC prediction equations in five periods of the day to the real 24-h urine iodine excretion (UIE), aiming for providing the evidence of individual iodine status assessment and promoting the study of the relationship between individual iodine status and diseases.

## Methods

### Participants

126 healthy adults aged 18-to-59-year olds were recruited through an advertisement from May to September 2016. Data on age and sex was collected. All spot urine and 24-h urine samples over a 24-h period were collected.

People included in this study were based on the following criteria: (1) non- pregnant and breastfeeding women; (2) no history of thyroid diseases; (3) no recent (within 6 months) use of iodine contrast agents or amiodarone medications; (4) no severe infectious disorders, chronic diseases, renal or other systemic diseases; (5) long-term residency (longer than six months) in Fujian Province. And the exclusion criteria were: (1) pregnant and lactating women; (2) recent (within 6 months) coronary angiography, endoscopic retrograde cholangiopancreatography (ERCP), and other use of iodinated contrast agent and amiodarone drugs; (3) having renal dysfunction and (or) other major diseases; (4) with mental diseases; (5) having cognitive disorders; (6) not living in Fujian Province for six months.

### Urine samples collection

Participants were given a uniform urine collection bag, a sterile plastic urine collection tube with a handle for dipping, and clear instructions for 24-h urine collection. Participants were required to record the start and finish times. All the participants were informed about the collection methods and announcements. The urine was firstly collected after the first urination in the early morning; then it was collected till the first urination on the next morning, lasting for 24 h. Each voiding time was recorded. There were five periods of the day for urine collection: morning (after discarding the first void − 12:30), afternoon (12:31–17:30), evening (17:31–23:59), early morning (00:00–03:59), and fasting time (the first void collected the next morning after the longest duration of sleep) [26]. 24-h urinary creatinine excretion [27]was used as quality control for 24-h urine collection, and participants with urinary creatinine excretion of less than 75 per cent were eliminated across genders and ages [28].

### Laboratory analysis

All results were assayed in the Fujian Provincial Centre for Disease Control and Prevention, which satisfied the requirements of the quality control of the National Iodine Deficiency Disorders Reference Laboratory in China. Urine iodine concentration was assayed by Arsenic-Cerium Catalytic Spectrophotometry for Urine Determination (WS/T107-2016) [29], and urinary creatinine concentration was assayed applying urinary creatinine alkaline picric acid spectrophotometry (WS/T97-1996) [30].

The laboratory assaying process included quality control. The reference substances were used, and the standard curve's correlation coefficient had to be greater than 0.999.

### Equations of the estimated and measured 24-h urinary iodine excretion

For spot urine and fasting urine in five periods of the day, the six prediction equations were employed to estimate 24-h UIE in the same individual. The equations in Table 1 were used to estimate UIE.

**Table 1:** Equations of the estimated and measured 24-h urinary iodine excretion

Measure	Abbreviation	Calculation
Iodine: creatinine ratio (μg/g)	I/Cr	UIC (μg/L) / UCr (g/L)
Measured 24-h urine iodine excretion (μg/d)	Measured 24hUIE	24hUIC (μg/L) × 24 h urine (L)
Estimated 24-h urine iodine excretion^1^ (μg/d)	Estimated 24 h UIE^1^	I/Cr (μg/g) × Pr24hCr (g/d)
		Pr24hCr (g/d) = 24hUCr (g/L) × 24 h urine (L)
Estimated 24-h urine iodine excretion^2^ (μg/d) (Tanaka T, et al.[31])	Estimated 24 h UIE^2^	I/Cr (μg/g) × Pr24hCr (g/d)
		Pr24hCr (mg/d) = [(− 2.04 × age (year)] + [(14.89 × weight
		(kg)] + [16.14 × height (cm)]—2244.45
Estimated 24-h urine iodine excretion^3^ (μg/d) (Kawasaki T, et al.[32])	Estimated 24 h UIE^3^	I/Cr (μg/g) × Pr24hCr (g/d)
		Males:
		Pr24hCr (mg/d) = [− 12.63 × age (year)] + [15.12 × weight
		(kg)] + [7.39 × height (cm)]—79.9
		Females:
		Pr24hCr (mg/d) = [− 4.72 × age (year)] + [8.58 × weight (kg)]
		+ [5.09 × height (cm)]—74.5
Estimated 24-h urine iodine excretion^4^ (μg/d) (Mage, et al.[33])	Estimated 24 h UIE^4^	I/Cr (μg/g) × Pr24hCr (g/d)
		Males:
		Pr24hCr (mg/d) = 0.00179 × [140—age (year)] × [weight
		(kg)^1.5^ × height (cm)^0.5^] × [1 + 0.18 × A × [1.366–0.0159 ×
		BMI (kg/m^2^)].
		Females:
		Pr24hCr (mg/d) = 0.00163 × [140—age (year)] × [weight
		(kg)^1.5^ × height (cm)^0.5^] × [1 + 0.18 × A × [1.429–0.0198 ×
		BMI (kg/m^2^)]; where A is African American or black race = 1,
		other race = 0
Estimated 24-h urine iodine excretion^5^ (μg/d) (Knudsen N, et al.[27])	Estimated 24 h UIE^5^	I/Cr (μg/g) × Pr24hCr (g/d)
		Males:
		pr24hCr (g/d) = 1.74 g (25–49 years), 1.63 g (50–59 years)
		Females:
		pr24hCr (g/d) = 1.23 g (25–49 years), 1.15 g (50–59 years)
Estimated 24-h urine iodine excretion^6^ (µg/d) (IOM[34])	Estimated 24 h UIE^6^	iodine excretion (µg/d) = 0.9 × iodine intake (µg/d)
		iodine intake (µg/d) = spot UIC (µg/L)/ 0.92 × (0.0009 L · h^−1^
		· kg^−1^ · 24 h · d^−1^) × weight (kg)

*I,* Iodine; *Cr,* creatinine; *UIC,* urinary iodine concentration; *UIE,* urine iodine excretion; *Pr24hCr,* 24-h creatinine prediction

Stepwise forward linear regression analysis was used to model the Estimated 24 h UIE^2^

Regression analysis a regression equation was used to model the Estimated 24 h UIE^3^

Body surface area (BSA) was used to model the Estimated 24 h UIE^4^

The data from a large Belgian population study was used to model the Estimated 24 h UIE^5^

The Iowa State University method was used to model the Estimated 24 h UIE^6^

### Statistical analysis

The analysis was conducted by SPSS software (SPSS 20.0; IBM Corp, Armonk, NY, USA). The normality of data was checked by the Kolmogorov-Smirnoff test. Age, height and weight were presented as means ± SD for normally distributed data, and 24-h urine volume was presented as medians (95% CI). The difference between the estimated UIE and measured UIE (standard method) were examined by applying independent samples *t*-tests for normally distributed continuous variables, Mann–Whitney *U* tests for non-normally distributed continuous variables. The correlations between the estimated UIE and measured UIE (standard method) were tested by Pearson correlation for normally distributed data and Spearman correlation for non-normally distributed data. The consistency between the estimated UIE and measured UIE (standard method) was tested by a Bland–Altman plot. *P* < 0.05 was considered as significantly different.

Ethics approval was obtained from the Ethics Committee of Fujian Provincial Centers for Disease Control and Prevention (No. 2017002). Written informed consents were obtained from all the participants before their urine samples were collected.

## Results

### Demographic characteristics of the participants

A total of 126 healthy adults of which 59 males and 67 females were enrolled in this study. Age, height, weight, and 24-h urine volume were shown in Table 2.

**Table 2:** Demographic characteristics of 126 participants

Sex	Female (n = 67)	Male (n = 59)	Total (n = 126)
Age (year), mean ± SD	40.1 ± 11.83	37.2 ± 10.45	38.7 ± 11.25
Height (m), mean ± SD	1.60 ± 0.04	1.69 ± 0.05	1.64 ± 0.07
Weight (kg), mean ± SD	55.5 ± 7.47	67.3 ± 8.15	61.0 ± 9.74
24-h urine volume (L), IQR	2.14 (1.54, 2.68)	2.09 (1.45, 2.59)	2.09 (1.47, 2.62)

*SD,* standard deviation; *IQR,* inter-quartile range

### Comparison between the measured and the estimated 24-h urinary iodine excretion in different periods of the day

In this study, 121 spot urine samples were collected in the morning, 126 spot urine samples were collected in the afternoon, 125 spot urine samples were collected in the evening, and 48 spot urine samples were collected in the early morning, and 126 in the fasting time.

**Fig. 1:**
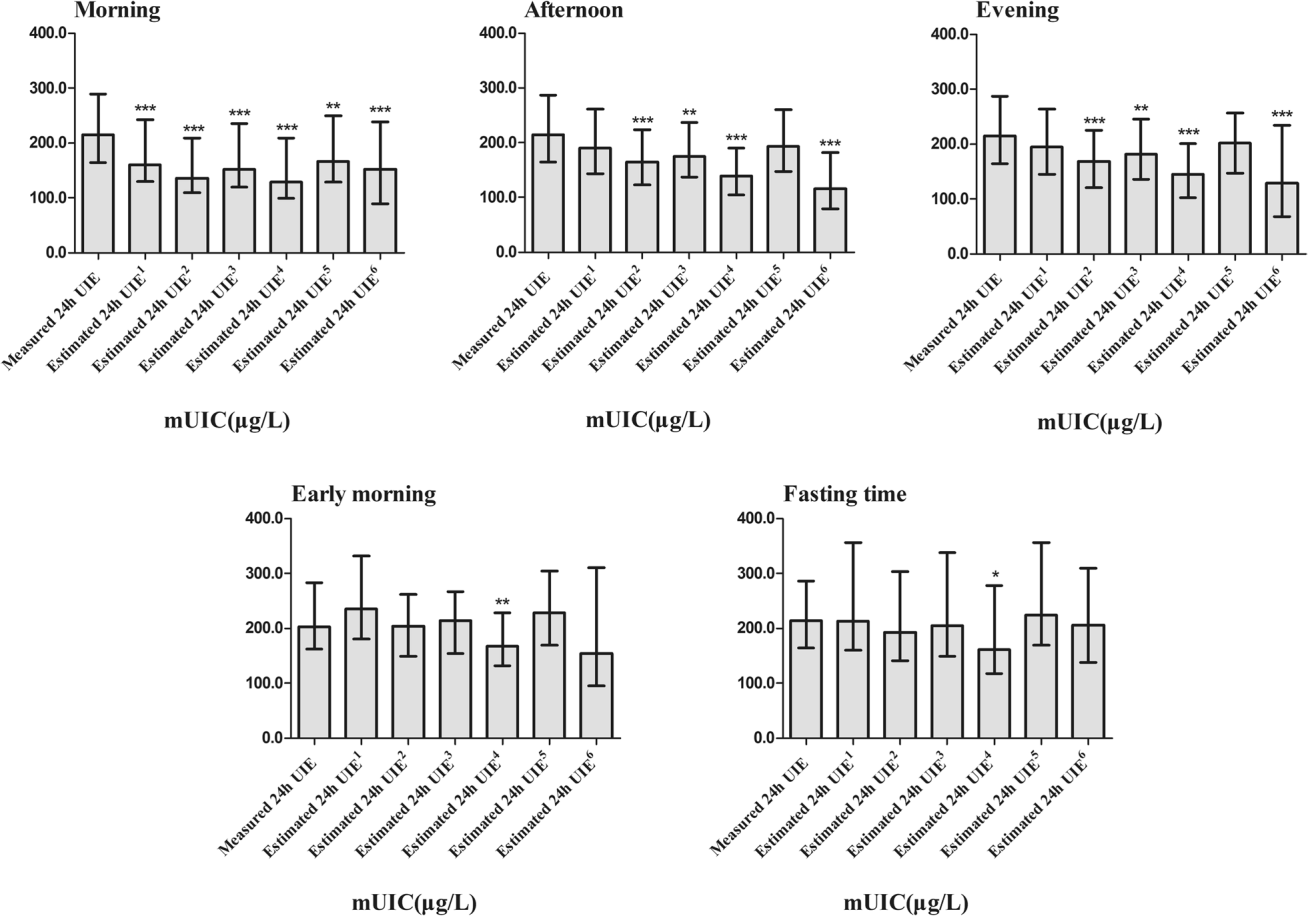
Difference between measured and estimated urinary iodine excretion in different periods of the day. *UIE,* urine iodine excretion; *mUIC,* median urinary iodine concentration; ^*^Estimated urinary iodine excretion was significantly different compared to the measured urinary iodine excretion (*P* < 0.05) by Spearman rank correlation analysis; ^**^Estimated urinary iodine excretion was significantly different compared to the measured urinary iodine excretion (*P* < 0.01) by Spearman rank correlation analysis; ^***^Estimated urinary iodine excretion was significantly different compared to the measured urinary iodine excretion (*P* < 0.001) by Spearman rank correlation analysis

The 24-h UIE of spot urine in the morning was estimated by Estimated 24 h UIE^1^ and Estimated 24 h UIE^5^, and there was a significant difference between measured and estimated UIE (*P* < 0.001). Similarly, there was no significant difference (*P* > 0.05) between measured and estimated 24-h UIE of fasting urine and spot urine in the afternoon, evening, and early morning, respectively. Estimated 24-h UIE^2^ and Estimated 24-h UIE^3^ were used to estimate 24-h UIE of spot urine in the morning, afternoon, and evening, and significant differences between measured and estimated results (*P* < 0.01 for all) were found. Likewise, the difference between measured and estimated 24-h UIE of fasting urine and spot urine in the early morning was non-significant (*P* > 0.05). In all periods of time, the Estimated UIE^4^ was significantly different from the measured ones (*P* < 0.05 for all). The difference between the measured iodine concentration and the estimated one was statistically significant (*P* < 0.001 for all) by Estimated 24-h UIE^6^ in the morning, afternoon, and evening. Similarly, the estimated 24-h UIE of fasting urine and spot urine in the early morning showed that the measured and estimated results were not significantly different (*P* > 0.05 for both) (Fig. 1).

### Correlation between the measured and estimated 24-h urinary iodine excretion in the different time periods of the day

The estimated UIE showed a significant linear correlation compared to the Measured 24-h UIE urine samples collected in all periods of the day (*P* < 0.01 for all). (Table 3).

**Table 3:** Correlation between the measured and estimated urinary iodine excretion in different periods of the day

	Measured 24 h UIE VS estimated 24 h UIE^1^	Measured 24 h UIE VS estimated 24 h UIE^2^	Measured 24 h UIE VS estimated 24 h UIE^3^	Measured 24 h UIE VS estimated 24 h UIE^4^	Measured 24 h UIE VS estimated 24 h UIE^5^	Measured 24 h UIE VS estimated 24 h UIE^6^
Morning	< 0.001^##^	< 0.001^##^	< 0.001^##^	< 0.001^##^	< 0.001^##^	< 0.001^##^
Afternoon	< 0.001^##^	< 0.001^##^	< 0.001^##^	< 0.001^##^	< 0.001^##^	0.001^#^
Evening	< 0.001^##^	< 0.001^##^	< 0.001^##^	< 0.001^##^	< 0.001^##^	< 0.001^##^
Early morning	< 0.001^##^	< 0.001^##^	< 0.001^##^	< 0.001^##^	< 0.001^##^	0.002^#^
Fasting time	< 0.001^##^	< 0.001^##^	< 0.001^##^	< 0.001^##^	< 0.001^##^	< 0.001^##^

*UIE,* urine iodine excretion;

^#^Estimated urinary iodine excretion was significantly different compared to measured urinary iodine excretion (*P* < 0.01) by Spearman rank correlation analysis;

^##^Estimated urinary iodine excretion was significantly different compared to measured urinary iodine excretion (*P* < 0.001) by Spearman rank correlation analysis

### Consistency between the measured and estimated 24-h urinary iodine excretion

With the exception of the morning, there was no significant difference but a strong correlation between the measured and estimated 24-h UIE in the different time periods. For all prediction equations except Estimated 24-h UIE^4^, fasting urine might be a choice to measure 24-h UIE.

**Fig. 2:**
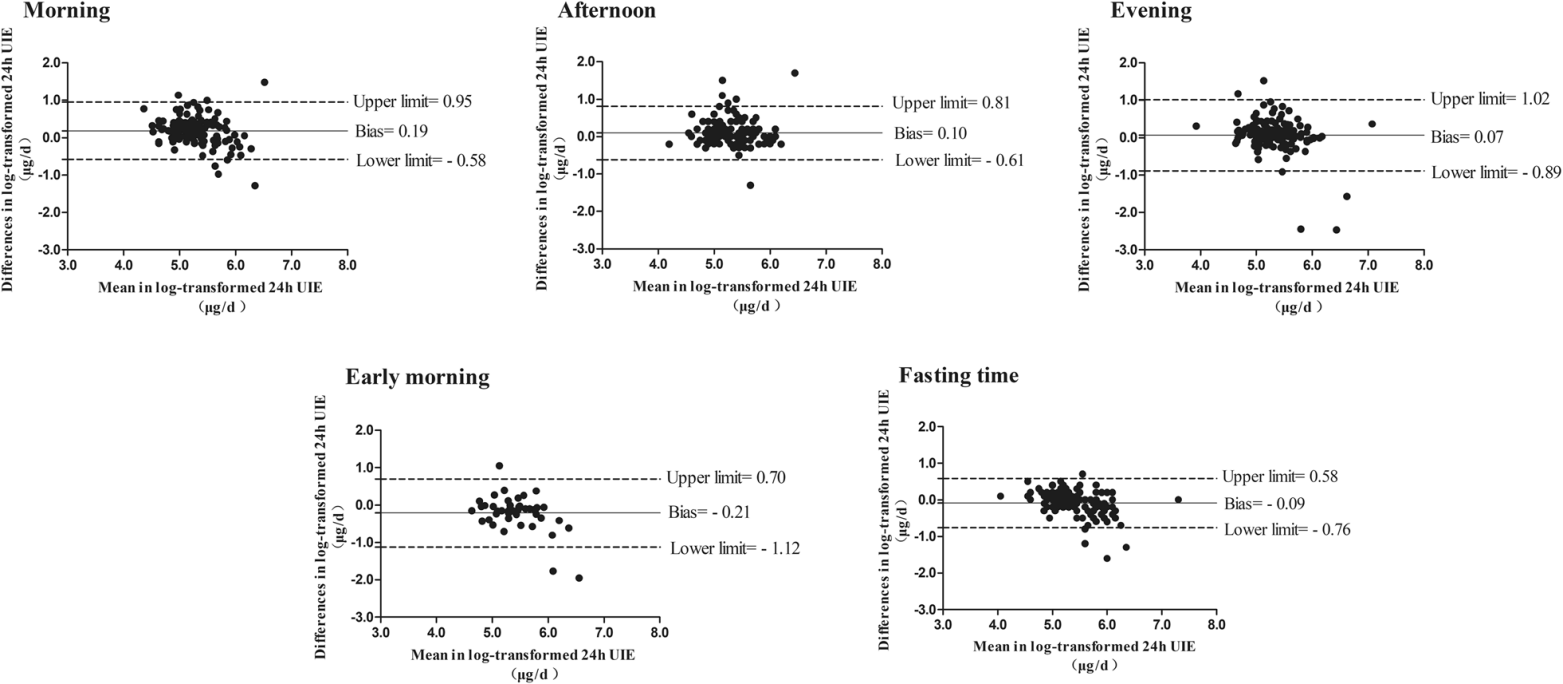
Consistency between log-transformed Estimated 24-h urinary iodine excretion^1^ and log-transformed Measured 24-h urinary iodine excretion in different periods of the day. UIE, urine iodine excretion; The X-axis is the mean of log-transformed estimated 24-h urinary iodine excretion^1^ and log-transformed measured 24-h urinary iodine excretion; The Y-axis is the difference between log-transformed estimated 24-h urinary iodine excretion^1^ and log-transformed measured 24-h urine iodine excretion; The solid black line represents the bias, and the dashed line represents the 95% range of consistency for the mean relative difference; Upper limit: upper 95% limit of consistency; lower limit: lower 95% limit of consistency

Hereby, Bland–Altman plots were also designed to evaluate the consistency between the measured and the estimated 24-h UIE. The bias of Estimated 24-h UIE^1^ varied from − 0.21 to 0.19 by urine sample collected time at the individual level. The high consistency occurred in the evening time period (bias = 0.07) and fasting time (bias = − 0.09) (Fig. 2).

**Fig. 3:**
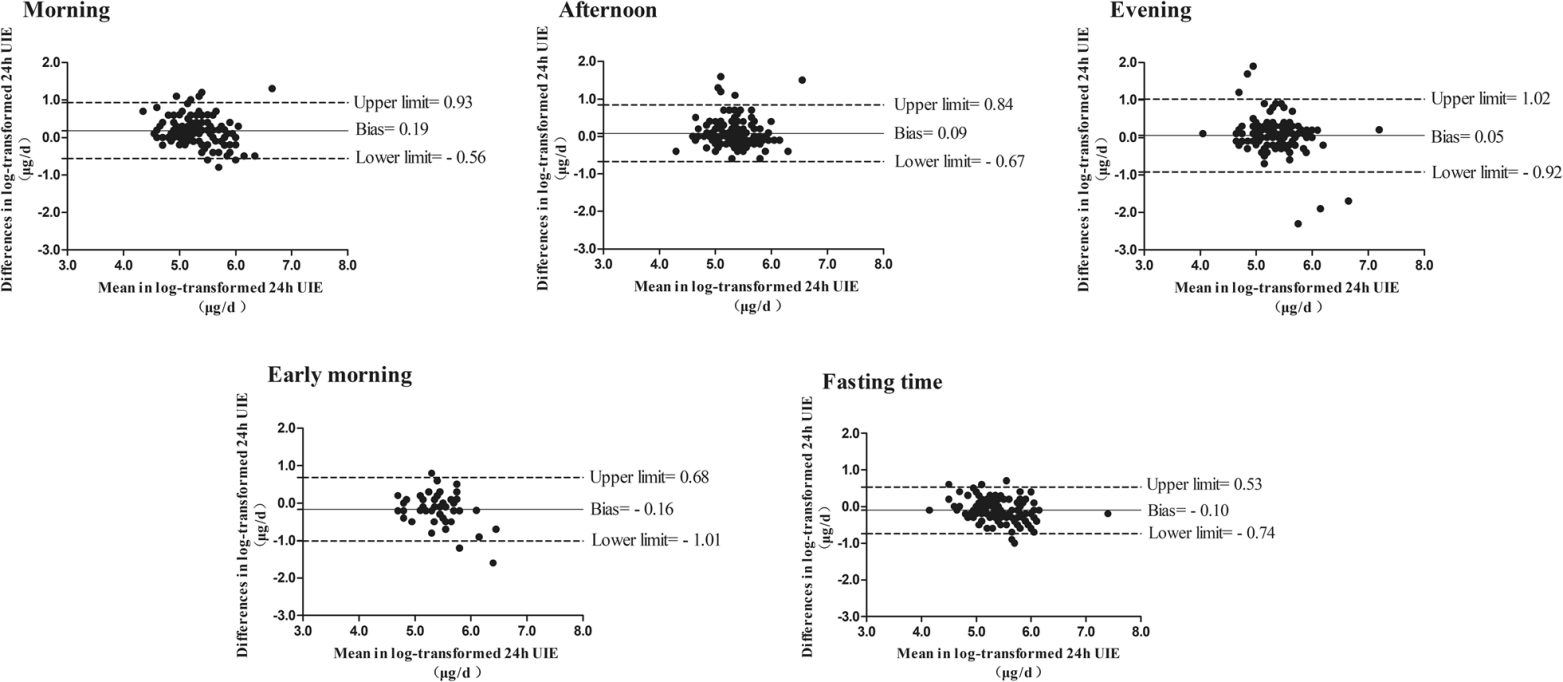
Consistency between log-transformed Estimated 24-h urinary iodine excretion^5^ and log-transformed Measured 24-h urinary iodine excretion in different periods of the day. *UIE,* urinary iodine excretion

We also discovered that the bias of Estimated 24-h UIE^5^ varied from − 0.16 to 0.19 in different periods of the day. As for spot urine, the estimated UIE showed a high consistency when compared to the measured ones in the afternoon (bias = 0.09) and evening (bias = 0.05) (Fig. 3).

**Fig. 4:**
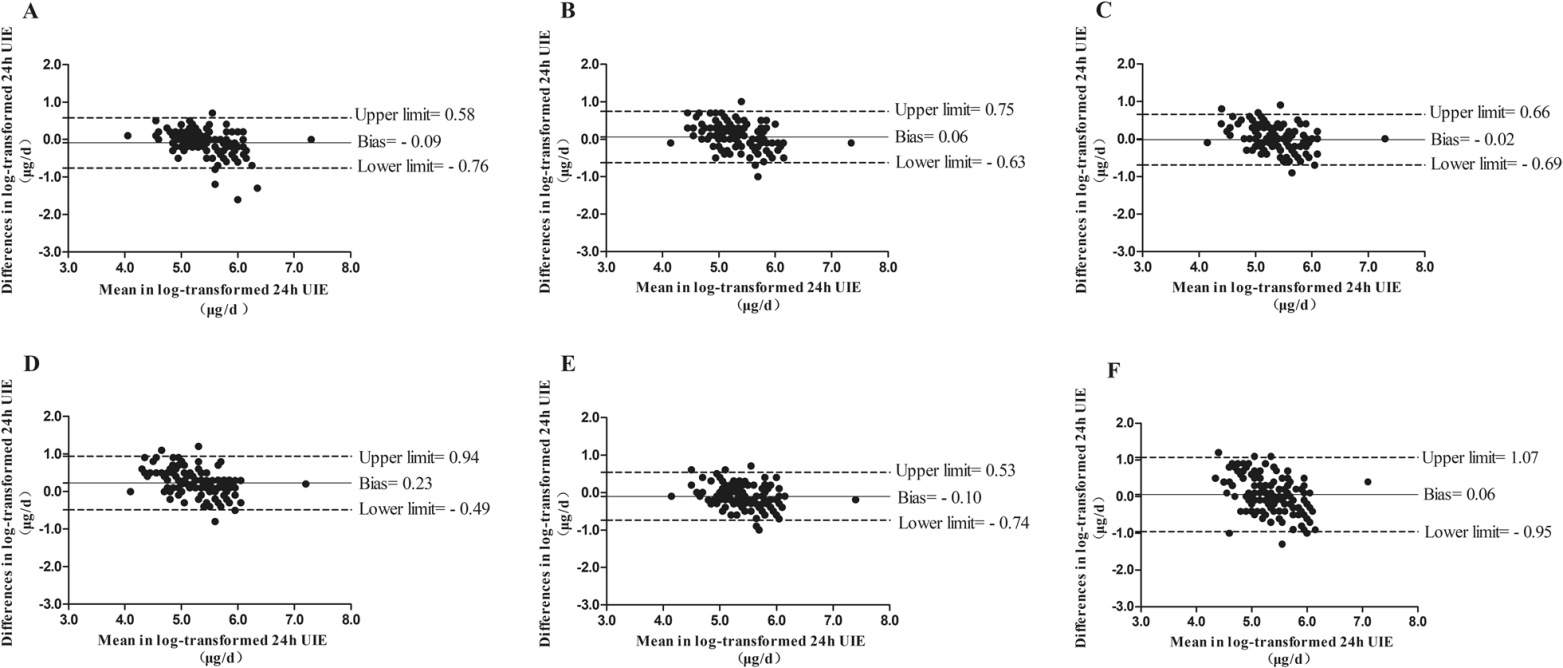
Consistency of the six estimated 24-h urinary iodine excretion equations and the measured 24-h urinary iodine excretion in fasting urine. *UIE,* urine iodine excretion. Estimated 24 h UIE^1^ for (**A**), Estimated 24 h UIE^2^ for (**B**), Estimated 24 h UIE^3^ for (**C**), Estimated 24 h UIE^4^ for (**D**), Estimated 24 h UIE^5^ for (**E**) and Estimated 24 h UIE^6^ for (**F**);

In the prediction of fasting urinary iodine excretion, Estimated 24-h UIE^3^ (bias = − 0.02) showed the best consistency while Estimated 24-h UIE^4^ (bias = 0.23)was the worst one, and Estimated 24-h UIE^1^ (bias = − 0.09), Estimated 24-h UIE^2^ (bias = 0.06), Estimated 24-h UIE^5^ (bias = − 0.10), and Estimated 24-h UIE^6^ (bias = 0.06) showed a relatively good consistency (Fig. 4).

## Discussion

The findings of our study demonstrated that Estimated 24-h UIE^5^ might be a choice to estimate 24-h UIE at all times except the morning. Fasting urine could be used to estimate 24-h UIE in several prediction equations. With the exception of Estimated 24-h UIE^4^, the other prediction equations by fasting urine are feasible in the prediction of 24-h UIE, especially Estimated 24-h UIE^3^.

Currently, there is no uniform method for individual iodine status assessment and each method has its limitations. The 24-h UIE, computed by multiplying the 24-h UIC and the 24-h urine volume, is the acknowledged as "gold standard" for individual iodine status assessment. Some researchers have proposed numerous equations to estimate individual 24-h UIE given that it is not easy to collect 24-h urine. Jakobsen J et al. verified the completeness of 24-h urine collection by comparing the concentration of para-aminobenzoic acid (PABA) in urine to tablets, but it may be harmful to people’s health [35]. To perform the quality control of the completeness of 24-h urine collection, we set a creatinine reference value at 75%, which ensured that the 24-h urine volume for all volunteers was entirely collected. The equation concerning 24-h urine volume seemed reliable due to its results, particularly the Measured 24-h UIE. Once it was reliable, the estimated equation which was compared to Measured 24-h UIE could be used in the evaluation of individual iodine status. Although it is easy for spot urine collection, there is little evidence to support spot urine as a way to reflect iodine intake over the course of 24 h [36]. As a consequence, some researchers have proposed a variety of prediction equations to estimate 24-h UIE [27, 31–33]. Some researches showed that multiplying I/Cr by estimated 24-h urine creatinine could be used to estimate 24-h UIE while others could not [37].

Moreover, UIE varies during the day, but it is controversial on the circadian pattern. UIC which was at the lowest level in the morning showed an upward trend from the morning to the evening; and spot urine in the afternoon can represent 24-h UIE, according to Als C and Vanacor R et al. [28, 38]. As for fasting urine, one article pointed out that the UIC in fasting urine samples was 10% lower compared to non-fasting urine samples [39]. Nonetheless, some investigations reported that UIC in spot samples do not have a similar tendency during the day [40]. Similarly, the results presented in Fig. 1 do not have an upward trend for all the participants, conversely, each participant had a different tendency.

Other researchers have chosen blood samples to assess individual iodine status [23–25]. Although the metabolites in blood are more stable compared to urine and more representative of iodine status over a longer period of time, blood collection presents many challenges, including the demand of the blood collection specialist, the consumption of needles and biochemical tubes during blood collection, and the prevention of contamination of blood products after collection. Blood collection is more time-consuming and complicated than urine collection in terms of input costs and output results. As a result, it is very cost-effective to assess individual urinary iodine status by urine.

According to our findings, the six prediction equations may have applicability in the assessment of individual iodine status. We still needed to employ 24-h urine volumes for calculation when using Estimated 24-h UIE^1^ albeit most periods of the day (except the morning) showed good consistency. In addition, there was a large difference in 24-h urine volume between males and females [41, 42]. Furthermore, there is currently no reference range for urine volume by sex in China. Despite the fact that Estimated 24 h UIE^2^ and Estimated 24 h UIE^3^ were proposed on the Japanese population, which means there seem no racial differences between the Chinese and Japanese populations. And these two equations can be used to estimate measured 24-h UIE in the early morning and fasting time. Figure 1 also showed that the 24-h UIE in any period could not be predicted by Estimated 24-h UIE^4^, which implied that iodine excretion in various groups varied considerably depending on ethnicity, social-economic status, and dietary patterns [43]. Estimated 24-h UIE^5^ was one of the prediction equations that may be suggested to estimate UIE in all periods except the morning. However, it was difficult to collect urine samples in the evening although they had the highest consistency. Estimated 24-h UIE^6^, as an equation for children UIE prediction, showed the infeasibility among adults, which verified the premise that only for the children population.

Similarly, estimated 24-h UIE in different periods of the day also showed inconsistent results. The prediction equation except Estimated 24-h UIE^4^ showed no significant difference between the early morning spot urine and fasting urine although they had a high correlation. It is not suggested to estimate by early morning spot urine since not all participants urinate during this period of the day, therefore, it is hard to assess iodine status for every person by collecting early morning spot urine. In addition, several researchers pointed that it was better to estimate 24-h UIE by fasting urine compared to other spot urine [44, 45]. The findings of this study also suggested that it was preferable to estimate 24-h UIE by fasting urine samples in most periods of the day. Also, The Bland–Altman diagram indicated that estimated 24-h UIE^3^ was the best for fasting urine estimation.

Salt iodization is a good way to get enough iodine in a consistent, convenient, and cost-effective way [46, 47]. We can benefit from this small investment and improve the quality of life for the population. A high-salt diet, on the other hand, is linked to hypertension. Currently, the Sydney Technical Consultation recommends that population health can be improved by salt reduction and iodine fortification strategies. While preventing iodine deficiency, it also protects against the harmful effects of salt on cardiovascular disease (CVD), especially hypertension [17]. In this study, the equations of creatinine corrected UIC can be used to evaluate individual iodine status and serve as the method in the prevention of CVD. Although the findings of the American Food and Drug Administration Total Diet Study suggest that cereals, milk, and cheese can be served as dietary iodine carriers [48], some researchers have found a negative association between dairy products consumption and hypertension among college students [16]. However, dairy products such as milk are only minor components in the Chinese diet, and dairy products cannot replace iodized salt in meeting the iodine needs of the Chinese people’s iodine intake. Iodine deficiency, on the other hand, is a determinant of adult thyroid diseases [49]. Iodine deficiency not only leads to iodine deficiency disorders, but also increases the risk of papillary and follicular thyroid cancer [50]. The risk of goitre rises with the increasing iodine levels [51]. As a result, establishing a method for individual iodine status is beneficial not only for determining individual iodine status but also for preventing CVD and thyroid diseases.

There are some strengths and limitations in this study. To our knowledge, this was the first time in China that individual iodine status in adults were estimated by equations with different creatinine-corrected urinary iodine concentrations at different times. Secondly, this study demonstrates that individual iodine status can be assessed by creatinine-corrected urinary iodine concentrations albeit single UIC has been previously used to assess iodine status among populations. For the limitation, firstly, currently, there is no reference range of creatinine for the Chinese population by age group, so Knudsen N's equation has its limited replication within the Chinese adults. Secondly, we had only 126 participants, which meant the sample size was not big enough. The external validity is uncertain since the participants were only recruited from Fujian Province instead of all the provinces in China. Lastly, the applicability for school-age children, pregnant women and lactating women were uncertaint since adults were the target population in our study.

## Conclusion

The application of the prediction equation proposed by Kawasaki T et al. was the best choice for the evaluation of individual iodine status in fasting urine. The prediction equation proposed by Knudsen N et al. is also suitable for the evaluation of individual iodine status if urine was collected in the non-morning period.

## Data Availability

Please contact author (Zhihui Chen) for data or material requests.

## Abbreviations

I: Iodine
Cr: Creatinine
UIC: Urinary iodine concentration
UIE: Urine iodine excretion
Pr24hCr: 24-Hour creatinine prediction
SD: Standard deviation
IQR: Inter-quartile range
mUIC: Median urinary iodine concentration
